# Squamous Vaginal Papillomatosis in Prepubertal Female Twins: A Case Report

**DOI:** 10.15388/Amed.2021.28.2.18

**Published:** 2021-12-22

**Authors:** Evelina Boreikaitė, Vytautas Bilius, Elžbieta Bumbul-Mazurek, Žana Bumbulienė

**Affiliations:** Clinic of Obstetrics and Gynecology, Institute of Clinical Medicine, Faculty of Medicine, Vilnius University, Vilnius University Hospital Santaros klinikos, Vilnius, Lithuania.; Clinic of Children Diseases, Institute of Clinical Medicine, Faculty of Medicine, Vilnius University, Vilnius University Hospital Santaros klinikos, Vilnius, Lithuania.; 1^st^ Department of Obstetrics and Gynecology Medical University of Warsaw, Warsaw, Poland; Clinic of Obstetrics and Gynecology, Institute of Clinical Medicine, Faculty of Medicine, Vilnius University, Vilnius University Hospital Santaros klinikos, Vilnius, Lithuania.

**Keywords:** vaginal papillomatosis, vulvar fibroepithelial polyp, female twins, prepubertal girls

## Abstract

This is the first case describing vaginal papillomatosis with a fibroepithelial polyp of the vulva in a prepubertal girl and vaginal papillomatosis in her twin sister. Parents contacted pediatric urologist regarding their eight-year-old daughter (twin A), who had a growth next to the external urethral meatus. The girl was referred to a pediatric surgeon. The exophytic 3 cm long structure with necrosis on top was found. After obtaining informed consent from girl parents, pediatric surgeon removed the exophytic structure and perform cystoscopy and vaginoscopy for possible changes in the bladder and vagina. Cystoscopy findings were normal. On vaginoscopy, numerous macroscopic papillomatous structures were identified on the cervix and vaginal walls. Vaginal biopsies were performed on the areas affected by papillomatosis. Histopathologic examination showed a fibroepithelial polyp with a central fibrovascular core covered by squamous epithelium and vaginal squamous papillomatosis. The decision was made to perform vaginoscopy on her twin sister (twin B), too. On vaginoscopy, solitary small vaginal papillomas were also found. In this case manifestation of vaginal papillomatosis in twins might have been influenced by inheritance and the same bacterial and viral environment.

## Abstract (lt)

**Makšties plokščialąstelinės papilomos dvynėms iki lytinio brendimo: klinikinis atvejis:**

**Santrauka. **Tai pirmasis klinikinis atvejis, kuriame aprašomos makšties plokščialąstelinės papilomos, diagnozuotos dvynėms iki lytinio brendimo, taip pat fibroepitelinis polipas nustatytas vienai iš dvynių. Tėvai kreipėsi į vaikų urologą dėl aštuonerių metų dukters (dvynės A), kuriai pastebėjo darinį šalia išorinės šlaplės angos. Mergaitė buvo nusiųsta vaikų chirurgo konsultacijai. Nustatyta egzofitinė 3 cm struktūra su nekroze viršutinėje dalyje. Gavus informuotą mergaičių tėvų sutikimą, vaikų chirurgas pašalino egzofitinę struktūrą ir atliko cistoskopiją bei vaginoskopiją dėl galimų šlapimo pūslės ir makšties pakitimų. Cistoskopijos metu patologijos nerasta. Atliekant vaginoskopiją, ant gimdos kaklelio ir makšties sienelių pastebėta daug makroskopinių papilinių struktūrų, biopsijos ištirtos histologiškai. Histologinis tyrimas nustatė fibroepitelinį polipą, padengtą plokščiu daugiasluoksniu ragėjančiu epiteliu, ir makšties plokščialąstelines papilomas. Nuspręsta vaginoskopiją atlikti ir jos dvynei seseriai (dvynei B), kuriai taip pat buvo rasta pavienių mažų makšties papilomų. Dvynių makšties plokščialąstelinės papilomatozės pasireiškimui galėjo turėti įtakos paveldimumas bei ta pati bakterinė ir virusinė infekcija.

**Raktiniai žodžiai: **makšties papilomatozė, vulvos fibroepitelinis polipas, dvynės, mergaitės iki lytinio brendimo

## Introduction

This is the first case describing vaginal papillomatosis with a fibroepithelial polyp of the vulva in a prepubertal girl and vaginal papillomatosis in her twin sister. Papillomatosis is a rare pathology of the female genitals. In the literature, most cases are associated with vulvar papillomatosis. The prevalence of vulvar vestibular papillomatosis is from 1% to 5% and is higher among young women [1,2], while the prevalence of vaginal squamous papillomatosis is unknown. According to literature, described cases occur in women ranging from 24 to 46 years [2-6]. There are no case reports available of this pathology in prepubertal girls. Fibroepithelial polyps of the vulva are diagnosed in older women, commonly aged 40 years, and are rarely seen before menarche or after menopause [7,8]. In literature we have found only four cases of fibroepithelial polyps in female newborns [9-12]. 

## Case report

**Figure 1.**
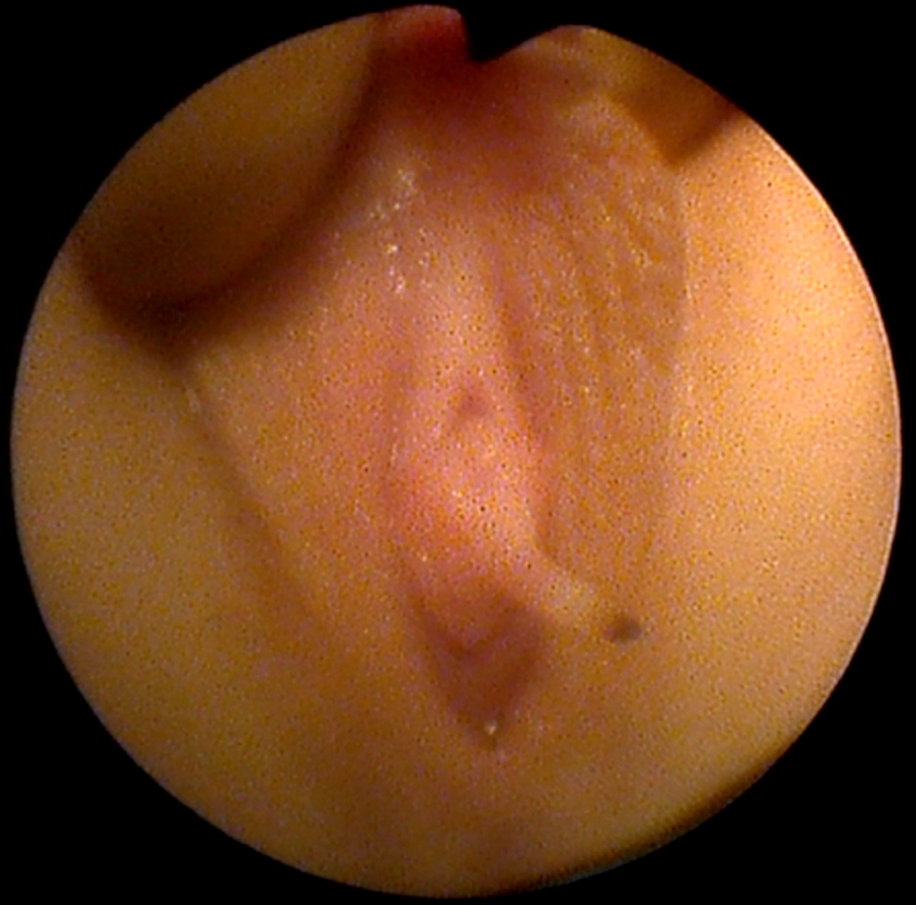
Fibroepithelial vulvar polyp next to the external urethral meatus inferior part (twin A).

**Figure 2.**
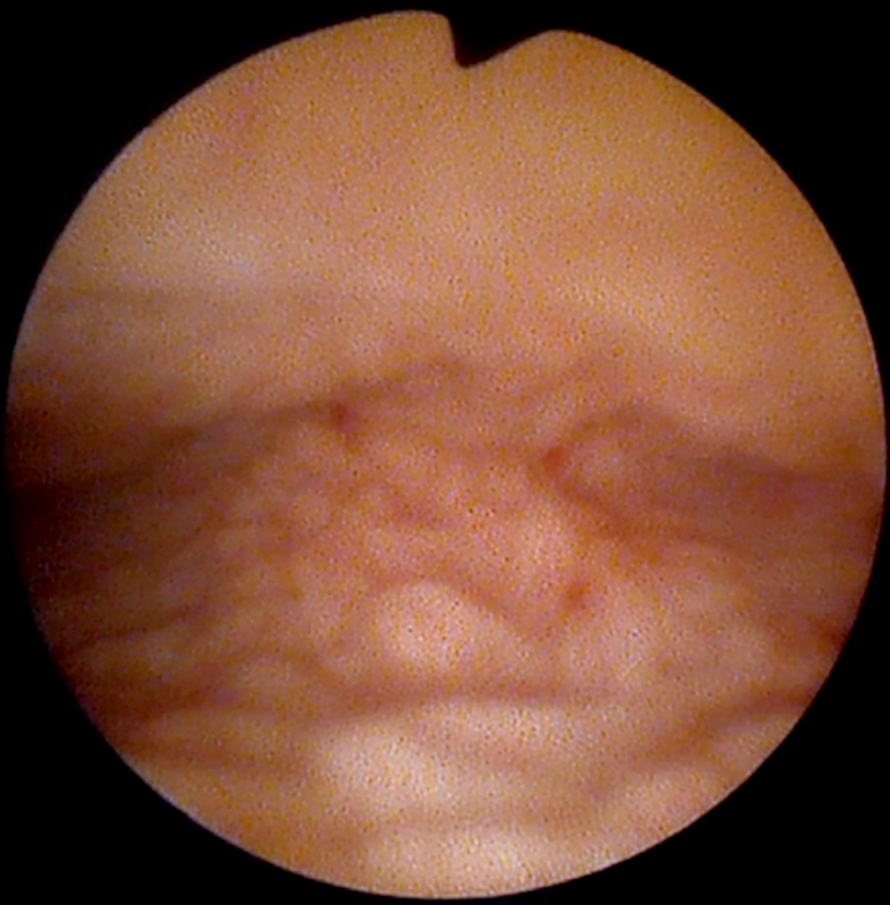
Vaginoscopy of twin A: papillomatous structures on vaginal walls.

Parents contacted pediatric urologist regarding their eight-year-old daughter (twin A), who had a growth next to the external urethral meatus. The girl was referred to a pediatric surgeon. The exophytic 3 cm long structure with necrosis on top was found next to the external urethral meatus (Figure 1). There were no other abnormal findings found on medical examination. Ultrasound imaging of the kidneys and pelvic organs also showed no pathology. After obtaining informed consent from the girl parents, during general anesthesia the pediatric surgeon removed the exophytic structure next to the external urethral meatus. It was also decided to perform cystoscopy and vaginoscopy for possible changes in the bladder and vagina. Cystoscopy findings were normal. On vaginoscopy, numerous macroscopic papillomatous structures were identified on the cervix and vaginal walls (Figure 2). Vaginal biopsies were performed in areas that were affected by papillomatosis. Histopathologic examination showed a fibroepithelial polyp with a central fibrovascular core covered by squamous epithelium and vaginal squamous papillomatosis. The specimens were analyzed for human papillomavirus DNA by real-time PCR assay (Anyplex II HPV28 Detection; Seegene) and were negative for 19 high risk HPV types (16,18,26,31,33,35,39,45,51,52,53,56,58,59,66,68,69,73,82) and 9 low risk HPV types (6,11,40,42,43,44,54,61,70). 

**Figure 3.**
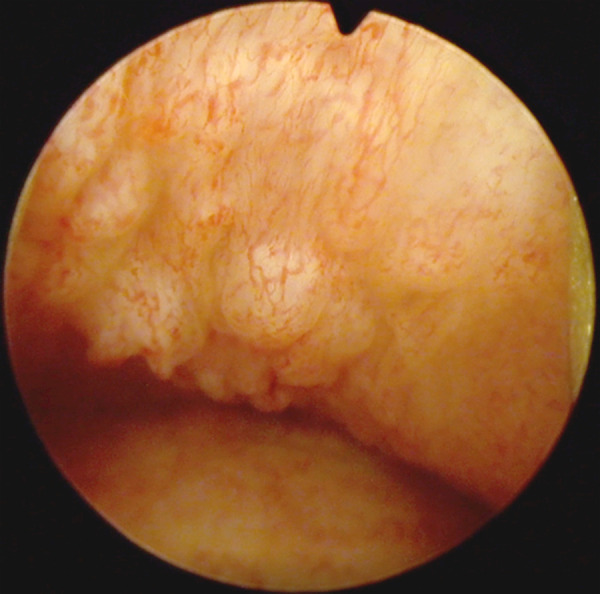
Vaginoscopy of twin B: solitary small vaginal papillomas.

Because the patient had a twin sister (twin B), the decision was made to perform vaginoscopy and cystoscopy for the second twin (twin B). Her cystoscopy was also normal. On vaginoscopy, solitary small vaginal papillomas were found (Fig. 3). The decision was made not to take a biopsy, and she was not analyzed for HPV DNA.

## Literature review

The majority of authors state that papillomatosis is a normal variant of the women genital mucosa [1,2,13,14]. In 1981, Altmeyer first described these lesions of the vulvar mucosa and named them pseudocondylomata of the vulva [15]. Up-to-date there are still many terminologies being used such as micropapillomatosis, papillae, pseudocondylomata, squamous papillomatosis, pruritic squamous papillomatosis, and hirsutoid papillomatosis. This might explain the unclear etiology of genital papillomatosis [1,6,15,16].

The clinical similarity of genital papillomatosis to warts has caused a disagreement about its etiology [5]. Results of most studies have shown that HPV is not associated with papillomatosis [6,14,17]. Although some authors state that the lesions are HPV-associated, [18] it is challenging for specialists to differentiate genital warts from genital papillomatosis, resulting in the wrong treatment choice. Five clinical features (Table 1), presented by Moyal-Barranco et al. could be used to diagnose and differentiate genital papillomatosis from other pathologies thus making additional procedures such as biopsies and HPV tests unnecessary [17].

**Table 1.** Clinical features of genital papillae.

Clinical features	Genital papillae	Condylomata acuminata
Distribution	Symmetrical, linear array	Random
Palpation	Soft	Firm
Color	Pink, same as adjacent mucosa	Pink, white, and red lesions often associated
Base	Bases of individual projections remain separate	Superficial filiform projections coalesce in a common base
Acetic acid test	No circumscribed whitening	Whitening in most cases

The majority of patients with genital papillomatosis are asymptomatic, whereas others might have complex symptoms (pruritus, pain or burning, dyspareunia) [6]. Growdon et al. noticed that HPV was diagnosed in symptomatic patients [18]. However, other authors have not determined this association [19].

Histology in cases of genital papillomatosis showed koilocytosis, which is a sign of HPV infection [20,21]. There are opinions that glycogen production has an impact on genital epithelium causing the formation of pales in the cell cytoplasm, which is easily misinterpreted as koilocytosis. Therefore, HPV can be over-diagnosed with histologic koilocytosis [6,16].

A fibroepithelial polyp of the vulva is a mucosal polypoid lesion, which consists of a connective tissue core and a cover of benign squamous epithelium [22]. It is known that the growth of these polyps is related to hormonal factors, hence it is important to ensure follow-up [8]. The treatment of vulvar polyps is excision, and its recurrence is rare [23]. Fibroepithelial polyps are benign lesions and, currently, there are no cases that have reported their transformation into malignant lesions [11].

## Conclusions

Vaginal squamous papillomatosis and vulvar fibroepithelial polyps are rare conditions in young girls. According to the literature, this is the first case of vaginal papillomatosis in twins. In our described case, the manifestation of papillomatosis in both girls might have been influenced by inheritance and the same bacterial and viral environment. There is a need for future studies confirming this relationship.
